# Antidote for the Bichloride of Mercury

**Published:** 1843-09

**Authors:** 


*Antidote for the Bichloride of Mercury.*—M. Orfila, having lately performed a series of experiments, with a view to determine the value of proto-sulphuret of iron as an antidote for corrosive sublimate, has published, in the "Journal de Chimie Medicale," the results at which he has arrived.

1. That the proto-sulphuret of iron completely destroys the poisonous properties of corrosive sublimate, if administered in sufficient quantity *immediately after* the ingestion of the poison.

2. That, like the other most approved antidotes for this poison, it is inefficacious, unless given within about ten or fifteen minutes, by which time the deleterious action of the poison is generally sufficiently powerful to cause death.

3. That, although the sulphuret decomposes the corrosive sublimate more powerfully and completely than *albumen*, and. therefore ought to be preferred in all cases where it can be administered *immediately*, or in *a very short time*, after the poison; yet it will almost always be found that *albumen* may be more advantageously resorted to than the sulphuret of iron, in consequence of the latter being kept only by the pharmaceutists, and much time being, therefore, lost before it can be administered, while white of egg mixed with water, which is everywhere to be obtained, may be given without delay.—*Prov. Med. Journ.*

